# Solubility Controlling Peptide Tags of Opposite Charges Generate a Bivalent Immune Response Against Dengue ED3 Serotypes 3 and 4

**DOI:** 10.3389/fimmu.2021.671590

**Published:** 2021-06-11

**Authors:** Nafsoon Rahman, Shiho Miura, Mami Okawa, Md. Golam Kibria, Mohammad Monirul Islam, Yutaka Kuroda

**Affiliations:** ^1^ Department of Biotechnology and Life Sciences, Graduate School of Engineering, Tokyo University of Agriculture and Technology, Tokyo, Japan; ^2^ Department of Biochemistry and Molecular Biology, University of Chittagong, Chittagong, Bangladesh

**Keywords:** SCP-tag (solubility controlling peptide tag), electrostatic interaction, sub-visible aggregates, dengue envelope protein domain 3 (ED3), immune response

## Abstract

We previously demonstrated that a protein’s immunogenicity could be substantially increased by attaching a hydrophobic solubility controlling peptide tag (SCP-tag) producing small sub-visible aggregates. Here, we report the oligomerization of Dengue envelop protein domain 3 (ED3), and consequently, its immunogenicity increase by mixing ED3s attached with SCP-tags of opposite charges at equimolar concentration. We used ED3 of serotype 3 (D3ED3) and serotype 4 (D4ED3), which are, respectively, moderately and poorly immunogenic, and their SCP tagged variants constructed by attaching either a C-termini 5-Aspartic acid (C5D) or a 5-Lysine (C5K) tag. Light scattering indicated that the isolated tagged ED3s remained monomeric, but mixing the C5D and C5K tagged ED3s at equimolar concentration generated sub-visible aggregates or oligomers of ~500 nm through electrostatic interaction. In addition, the oligomerized ED3s remained in a native-like state, as assessed by fluorescence spectroscopy and circular dichroism. The *in vivo* immunogenicity of the D3ED3 and D4ED3 oligomers generated by the charged tags increased by 5 and 16 fold, respectively. Furthermore, injection of heterotypic ED3 oligomers (D3C5D+D4C5K) induced an immune response against both D3ED3 and D4ED3 in 3 of 4 responsive mice, and the IgG titer of the bivalent anti-D3C5D-D4C5K sera was over 100 times higher than that generated by co-injecting the untagged D3ED3 and D4ED3 (D3+D4). Altogether, these observations suggest that SCP-tags could be used as a platform for producing a long-sought tetravalent dengue vaccine.

## Introduction

Proteins could be major vaccine candidates, but they are often questioned due to their poor immunogenicity (1); and the biopharmaceutical industry is continuously looking for novel, simple, and widely applicable methods for increasing a protein’s immunogenicity. Adjuvants are widely used to increase a protein’s immunogenicity (2), but the mechanisms behind the immunogenicity increase are not fully understood, and they can affect a protein’s biophysical properties (3). Thereby, adjuvants are not always a preferred choice in clinical practice (4). Besides adjuvants, biodegradable polymers (5) and nano-particles (6) are used to improve proteins’ immunogenicity. Furthermore, the fusion of lipoproteins, cytokines, or immunoglobulin domains are used to increase the immune response of the target protein (7, 8). To date, virus-like particle (VLP) is a recent strategy for enhancing a protein’s immunogenicity (9), which acts by presenting multiple copies of antigenic-epitopes on its surface (10, 11). Similarly, protein oligomerization can also produce repetitive antigens (12, 13) and thus increase a protein’s immunogenicity (14–16).

DENV is a flavivirus with four identified serotypes (DENV1-4). DENV’s single-stranded RNA encodes ten genes: The capsid (C), pre-membrane (prM), envelope (E), and seven nonstructural (NS) proteins (17). In particular, domain three of the E protein (ED3) is involved in the attachments of DENV to the host cell, and it is the major target of neutralizing antibodies (18, 19). Moreover, we previously showed that ED3 tagged with a hydrophobic 4-Ile tag generates a long-lasting immune response with T-cell memory in mice models (20). ED3 is thus a strong candidate for a protein-based dengue vaccine (18, 19, 21).

However, for proper protection against DENV, a tetravalent vaccine that ensures a high and equi-level immune response against all four serotypes is necessary because a heterotypic infection may lead to various complications such as Dengue Shock Syndrome (DSS) and Dengue Hemorrhagic Fever (DHF), reportedly through a mechanism known as Antibody Dependent Enhancement (ADE) (22). Unfortunately, though all four DENV serotypes’ ED3 have a high sequence (70-88%) and structural similarity (Root mean square deviation of 0.5-1Å) (23), they are not equally immunogenic. Thus, co-injecting ED3 from the four serotypes does not induce an equal immune response against all four DENV’s serotypes (24), and will thus not provide a tetravalent vaccine candidate that is actively investigated.

In previous reports, we showed that hydrophobic solubility controlling peptide tags (SCP-tags) (25–27) attached to the C-terminus of ED3 of DENV3 (D3ED3; 105 residues, 11.46 kDa) can produce small oligomers and thus increase a protein’s immunogenicity with a T-cell-dependent activation of the B-cells (20). This technique is based on the observation that the immunogenicity of a protein is strongly connected with its biophysical characteristics and aggregative properties (28–32). An essential benefit of the SCP-tag is its ability to generate sub-visible protein-specific aggregates in a highly reproducible and stable way (20, 25, 27) and thus increase the immune response in a fairly well-controlled manner (33). Therefore, in this work, we examined the immune response generated by injecting either homo or heterotypic ED3 oligomers produced by mixing ED3s tagged with two hydrophilic SCP-tags made of oppositely charged residues [5-Aspartic (C5D) and 5-Lysine (C5K) residues (25, 34–36)]. As a result, ED3 oligomerized with charged tags indeed increased the immunogenicity of the mildly immunogenic D3ED3 and the poorly immunogenic D4ED3 (103 residues, 11.13 kDa). Furthermore, by mixing D3ED3 and D4ED3 attached with oppositely charged tags, a bivalent anti-D3ED3-D4ED3 immune response was generated in 3 out of 4 mice, suggesting that oligomerization using charged SCP-tags could provide a biotechnological basis for the long-sought tetravalent dengue vaccine.

## Material and Methods

### Mutant Design, Protein Expression and Purification

**Figure 1:**
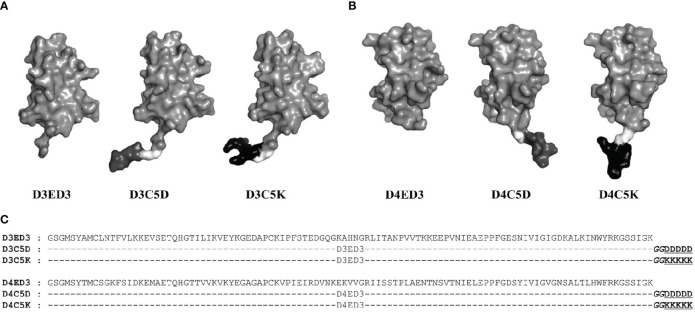
Sequences and schematic structures of the SCP-tagged ED3 variants. Surface models of the tagged **(A)** D3ED3 and **(B)** D4ED3 variants generated using the crystal structures of 3ED3 (3vtt.pdb) (50) and 4ED3 (3we1.pdb) (23) respectively, as templates. In short, we added the tag residues to the template structures using COOT (51) and MODELLER, assuming that the backbone structures remain unchanged. The pictures were generated using PyMOL (52). Glycine spacer is shown in light grey, the Aspartic and Lysine tags are shown in dark grey and black, respectively. **(C)** The sequences of D3ED3 and D4ED3 were obtained from UniProt (37,38). The 5-residue SCP-tag sequence, attached at the C-terminus of D3ED3 or D4ED3, is underlined and consists of two Glycines (italic), used as a spacer between the host protein and the tag.

The sequences of D3ED3 and D4ED3 were retrieved from UniProt (37, 38). The genes encoding ED3s were artificially synthesized and cloned into a pET15b vector (Novagen) at the endonuclease NdeI and BamH1 sites, as previously described (23, 39). The nucleotide sequences encoding the SCP-tags were added at the C-terminus of ED3 by QuikChange (Stratagene, USA) site-directed mutagenesis, and all sequences were confirmed by DNA sequencing. SCP-tagged ED3 variants were named according to the number and type of amino acids added to the C-terminus of D3ED3 and D4ED3 (20) (**Figure 1**). For example, D3C5D stands for a D3ED3 with five aspartic acids added after two glycines acting as a spacer between the tag and C-terminus of D3ED3.

The ED3 variants, namely, D3ED3, D4ED3, D3C5D, D3C5K, D4C5D, and D4C5K, were overexpressed in *E. coli* JM109 (DE3) pLysS as inclusion bodies. Protein expression was induced by the addition of 1.0 mM IPTG at OD590=0.5-0.6. The proteins were purified using Ni-NTA (Wako, Japan) in 6M guanidine-HCl (20, 43), refolded through dialysis, and the proteins in the soluble fraction were collected. The His-tag was cleaved by thrombin proteolysis, and the proteins were purified by a second passage through the Ni-NTA column, followed by reverse-phase HPLC (43). Protein identities were confirmed by analytical HPLC and MALDI-TOF mass spectrometry (Bruker Daltonics, autoflex III), and the lyophilized powders were stored at -30°C.

### Sample Preparation

Samples for all biophysical and immunization experiments were prepared as follows: The lyophilized protein powders were first dissolved in MilliQ water (Millipore A10 ultra-pure water purifier, EMD Millipore, Germany) at 1mg/mL, and the samples were centrifuged at 20,000xg for 20 minutes at 4°C to remove any aggregates that might have accumulated during sample preparation. The supernatants containing soluble proteins were aliquoted into 1mg/mL stock solutions.

We prepared two homo-serotypic combinations, (D3C5D+D3C5K and D4C5D+D4C5K), and two hetero-serotypic combinations (D3C5D+D4C5K and D3C5K+D4C5D). In order to generate the aggregates, C5D and C5K tagged proteins were mixed at a 1:1 ratio to yield a final protein concentration of 0.3 mg/mL in phosphate-buffered saline (PBS, pH 7.4), phosphate buffer (PB, pH 7.0) and Tris-HCl (pH 8.8). For example- D3C5D and D4C5K proteins were mixed at 0.15 mg/mL concentration yielding a total ED3 concentration of 0.3 mg/mL (0.15 + 0.15 mg/mL). The sample was named D3C5D+D4C5K. As a control, the untagged D3ED3 and D4ED3 proteins were mixed in the same way, and the formulation was termed D3+D4. The samples were incubated for 20 minutes at 25°C to generate aggregates. Monomeric ED3s were prepared in the same way. In brief, lyophilized protein powders were reconstituted at 0.3 mg/mL concentrations in PBS or PB and incubated for 20 minutes at 25°C. The final concentrations of ED3 molecules were identical (0.3 mg/mL) regardless of the fraction of homo-, hetero-, and monomeric proteins in the sample. In all cases, the final protein concentration was confirmed by measuring the extinction coefficient (A_280_) using Nanodrop-2000 (Thermo Fisher Scientific, USA).

### Biophysical and Spectroscopic Measurements

The hydrodynamic radii (*R*_h_) of the individual SCP-tagged ED3 variants and D3C5D+D4C5K aggregates were measured at 25° and 37°C by dynamic light scattering (DLS) (44) on a Zetasizer Nano-S (Malvern, UK). D3C5K+D4C5D formed visible aggregates (precipitates) and no biophysical measurements could be performed. For the other variants, three independent readings were recorded for calculating the average *R*_h_ from the number distributions using the Stokes-Einstein equation (45). The presence of sub-visible aggregates was also monitored by static light scattering (SLS) (46) at a wavelength of 600 nm using an FP-8500 spectrofluorometer (JASCO, Japan) and using a quartz cuvette with a 3 mm optical path length. Each SLS measurement was repeated three times, and the values were averaged.

The secondary structure contents were characterized by far-UV circular dichroism (CD) spectroscopy using a JASCO J820 CD spectropolarimeter (JASCO, Japan), and spectra were measured using a 2 mm optical path length quartz cuvette (20, 47). Trp-fluorescence spectra with λ_ex_ 295 nm were measured on a JASCO FP-8500 spectrofluorometer using a quartz cuvette with a 3 mm optical path length in order to examine the conformational stability of the ED3s (20, 23). Both measurements were conducted at a final protein concentration of 0.3 mg/mL at 25° and 37°C.

### Immunization Experiments

Four-week-old female mice (Jcl : ICR, CLEA, Japan) were used for the immunization experiments (**Supplementary Table S2**). Control mice were injected with PBS only. The D3ED3, D4ED3, and combinations of ED3s were formulated (see sample preparation section) and administrated at a final concentration of 30 µg/dose (100 µL/mice) as previously described (20). No adjuvant was used because the IgG antibody level was sufficiently increased by the sub-visible aggregates without a need for external adjuvant (20, 27). Besides, the aim of our study was to examine the effects of sub-visible aggregates on immunogenicity, *per see*, without external factors.

The samples were injected subcutaneously five times at weekly intervals. Note that, in addition to the aforementioned DLS measurements, we monitored the *R*_h_s just before injection by taking an aliquot of the injecting sample. Dose-dependent (doses 1-5) anti-D3ED3 and anti-D4ED3 antibody titers (IgGs) were measured three days after each inoculation using tail-bleed sera by enzyme-linked immunosorbent assay (ELISA).

After the fifth dose, the mice were kept untreated for seven weeks to assess their long-term IgG titers. During this period, the serum IgG levels were monitored weekly using the tail-bleed sera. On the seventh week, a sixth dose was injected, all mice were sacrificed after a week, and blood samples were collected from the heart, centrifuged, and the sera were preserved at -30°C. All of the animal experiments were performed in compliance with the Tokyo University of Agriculture and Technology (TUAT) review panel for animal experimentation and Japanese governmental regulations.

### ELISA

ELISA was carried out as previously described (20, 33). In short, anti-ED3 IgG levels were evaluated using the untagged D3ED3 and D4ED3 (2.5 µg/mL in PBS) as coating antigens on a 96-well microtiter plate (TPP microtiter plates, Japan). Anti-ED3 sera were applied to the PBS-washed wells at an initial dilution of 1:50 for the tail-bleed samples. Plates were then incubated at 37°C for 2 hours. In all ELISA plates, previously developed anti-dengue-ED3 (20) and anti-BPTI-19A sera (33) were used as positive and negative controls, respectively.

After washing the plates thrice with PBS-0.05% Tween-20, each well received a 100 μL of anti-mouse IgG HRP conjugate (Thermo Fisher Scientific, USA) at 1:3000 dilution in 0.1% BSA-PBS-Tween-20 and incubated at 37°C for 1.5 hours. As a substrate, O-phenyl Di-amine (OPD) was added. The color intensities were measured at 492 nm using a microplate reader (SH-9000 Lab, Hitachi High-Tech Science, Japan) immediately after stopping the reaction with 1 N sulfuric acid (50 µL/well). Antibody titers were calculated from the power fitting of OD_492nm_ versus the reciprocal of the antisera dilution using a cutoff of OD_492nm_ = 0.1 above the background values. The values were averaged over the number of mice (*n*) in the respective groups.

### Cell Surface CD Marker Analysis

Experimental samples were prepared according to our reported protocol (20). In short, single-cell suspension of mice splenocyte was prepared in FACS (Fluorescence Activated cell Sorter) buffer [PBS supplemented with 2% FBS (Fetal Bovine Serum), 1 mM EDTA, and 0.1% sodium azide]. Afterward, 1 X RBC lysis buffer (0.15 M ammonium chloride, 10 mM potassium bicarbonate, 0.1 mM EDTA) was used to lysis the red blood cells (RBCs). Furthermore, 1 million splenocyte cells in 100 µL pre-cooled FACS buffer were surface stained with different fluorescent labeled antibodies according to the manufacture’s guidelines. To study the CD4 T-lymphocytes, cells were stained with anti-CD3-Pcy5, CD4-Pcy7, CD44-FITC, and CD62L-PE-conjugated antibodies in one tube, and for CD8 T- lymphocytes, cells were stained with anti-CD3-Pcy5, CD8-Pcy7, CD44-FITC, and CD62L-PE-conjugated antibodies in another tube (0.2 μg of antibodies/100 μL) for 30 minutes in the dark. Unbound excess conjugated antibodies were removed by centrifugation, and the cells were resuspended in 500 μL of FACS buffer. The data were collected using a CytoFlex flow-cytometer (Beckman Coulter, USA).

## Results

### Biophysical Characterization of Sub-Visible Aggregates of D3ED3 and D4ED3 Variants

**Figure 2:**
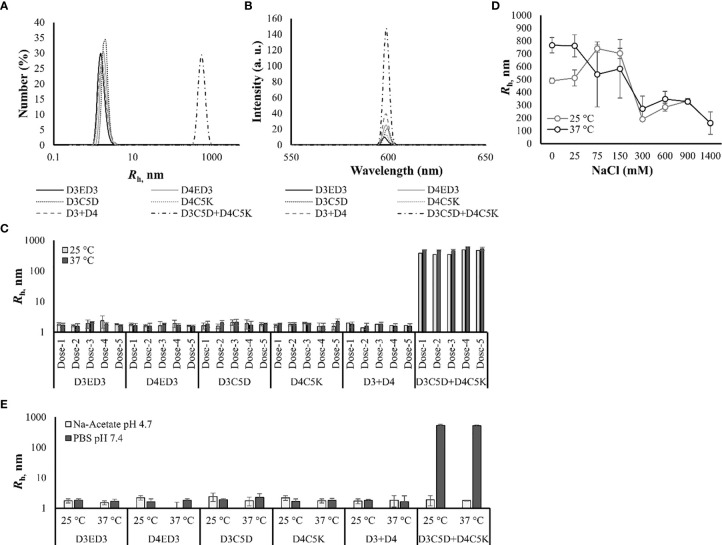
Sub-visible aggregates’ sizes measured by DLS and SLS. **(A)** DLS spectra of the size distribution number (%) at 37°C. **(B)** SLS spectra measured at 37°C. **(C)** Hydrodynamic radii of the tagged and untagged ED3s measured just before immunization from dose-1 to dose-4 at 25° and 37°C. **(D)** Salt-concentration [0-1400 mM] dependent hydrodynamic radius of D3C5D+D4C5K formulated in 10 mM PB, pH 7.0. **(E)** Hydrodynamic radii of D3ED3, D4ED3, D3C5D, D4C5K, D3+D4 and D3C5D+D4C5K when formulated in 10 mM Na-acetate buffer, pH 4.7 at 25° and 37°C. The *R*_h_ were computed from DLS’s number spectra. Proteins were formulated at a final concentration of 0.3 mg/mL in PBS pH, 7.4 for all measurements. Values are averaged over three independent measurements and three accumulations. Line symbols are explained within the panels.

The influence of C5D and C5K tags on the formation of sub-visible aggregates were examined by dynamic light scattering (DLS) and static light scattering (SLS) at 25° and 37°C. The hydrodynamic radii of the untagged D3ED3, D4ED3, and tagged ED3s ranged from 1.6-2.4 nm (**Figures 2A, C**, and **Table 1**), strongly suggesting that the untagged ED3s, as well as the un-mixed tagged ED3s, did not aggregate (**Supplementary Figures S1A, B** and **Table 1**).

**Table 1:** Hydrodynamic radii (*R*_h_) of sub-visible aggregates of the tagged ED3s.

Protein identities	Temperatures	*R*_h_ (nm)
**D3ED3**	25°C	1.8 ± 0.2
	37°C	1.7 ± 0.3
**D4ED3**	25°C	1.6 ± 0.4
	37°C	1.8 ± 0.2
**D3+D4**	25°C	1.8 ± 0.1
	37°C	1.7 ± .04
**D3C5D**	25°C	1.9 ± 0.2
	37°C	1.8 ± 0.2
**D3C5K**	25°C	1.9 ± 0.3
	37°C	2.4 ± 0.2
**D3C5D+D3C5K**	25°C	743 ± 63
	37°C	596 ± 19
**D4C5D**	25°C	1.8 ± 0.3
	37°C	2.1 ± 0.3
**D4C5K**	25°C	1.6 ± 0.2
	37°C	1.8 ± 0.1
**D4C5D+D4C5K**	25°C	294 ± 17
	37°C	324 ± 18
**D3C5D+D4C5D**	25°C	1.6 ± 0.4
	37°C	1.9 ± 0.3
**D3C5K+D4C5K**	25°C	2.7 ± 0.2
	37°C	2.8 ± 0.3
**D3C5D+D4C5K**	25°C	535.6 ± 45
	37°C	522.3 ± 34

All proteins were formulated at a final concentration of 0.3 mg/mL in PBS except D3C5D+D3C5K and D4C5D+D4C5K, which were formulated in phosphate buffer (PB, pH 7.0) for the formation of aggregates. D3C5D+D4C5D and D3C5K+D4C5K were not immunized in mice but R_h_s were measured in order to understand the interaction between same charged tags. All R_h_ values are calculated from the number distributions using the Stokes-Einstein equation, and they are the average of three independent measurements measured before each round of immunization. The errors are standard deviations (SD).

On the other hand, mixing ED3 tagged with residues of opposite charges produced, as we expected, sub-visible aggregates of about 500 nm, which were stable over time (**Supplementary Figures S1C, D**). In particular, D3C5D and D4C5K mixed at an equimolar concentration (D3C5D+D4C5K) generated submicron aggregates with *R*_h_ of ~522 nm at 37°C (**Table 1** and **Figures 2A, C**) and showed an increased SLS signal (**Figure 2B** and **Supplementary Figure S1B**). To date, no aggregates were detected in sodium-acetate buffer (pH 4.7) (**Figure 2E**), where the aspartic acid side chains lose their negative charges, and the electrostatic interactions disappear. In addition, the aggregate’s size decreased at high salt concentrations, further corroborating our hypothesis that the aggregates originated from the electrostatic attraction between the C5D and C5K tags (**Figure 2D**).

**Figure 3:**
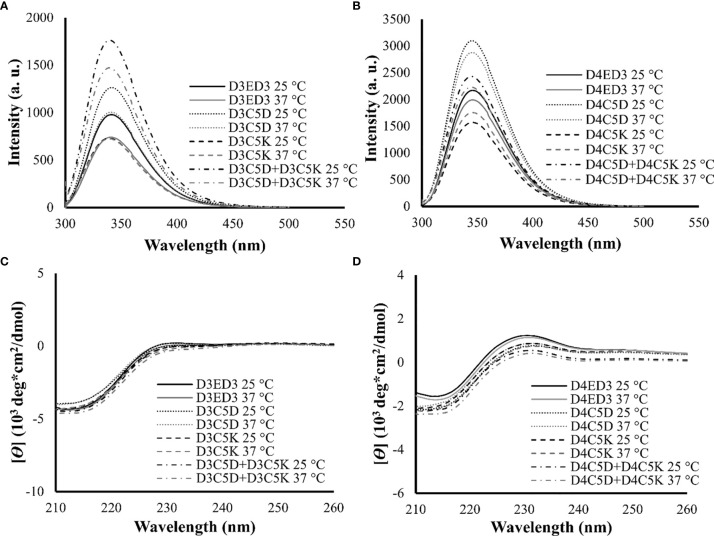
Structural characterization of SCP-tag induced sub-visible aggregates of homotypic ED3s by fluorescence and CD measurements. Trp-fluorescence spectra of **(A)** D3ED3 and **(B)** D4ED3 vatiants and CD spectra of **(C)** D3ED3 and **(D)** D4ED3 vatiants measured at 25° and 37°C. All protein samples were formulated at a final concentration of 0.3 mg/mL in PBS, pH 7.4 except D3C5D+D3C5K and D4C5D+D4C5K, which were formulated in phosphate buffer (PB, pH 7.0). Three accumulations were taken for each measurement. Line symbols are explained within the panels

**Figure 4:**
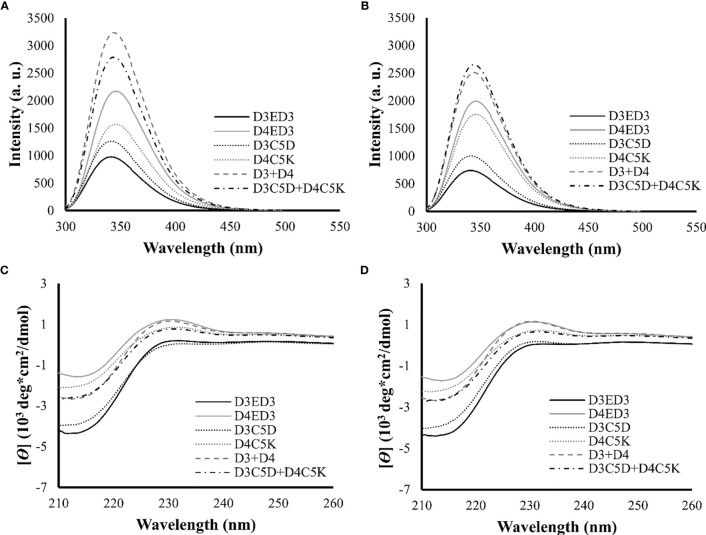
Structural characterization of SCP-tag induced sub-visible aggregates of heterotypic ED3s by fluorescence and CD measurements. Trp-fluorescence spectra of D3ED3, D4ED3, D3C5D, D4C5K, D3+D4 and D3C5D+D4C5K at **(A)** 25°C and **(B)** 37°C. CD spectra of the tagged and untagged variants as well as D3+D4 and D3C5D+D4C5K **(C)** 25°C and **(D)** 37°C. All protein samples were formulated in PBS, pH 7.4, at a final concentration of 0.3 mg/mL. Three accumulations were taken for each measurement. Line symbols are explained within the panels.

We assessed the impacts of the SCP-tags on the conformational stability of ED3 tagged variants and their aggregates using Trp-fluorescence and circular dichroism (CD) at 25° and 37°C. The SCP-tagged ED3 variants maintained their native-like conformations as assessed by Trp-fluorescence (**Figures 3A, B**) and CD (**Figures 3C, D**) at 25° and 37°C. Furthermore, ED3s attached to oppositely charged SCP-tags oligomerized in a native-like conformation (**Figure 4**) when mixed. Namely, Trp-fluorescence (48) (**Figures 4A, B**) and CD spectra (**Figures 4C, D**) of the mixed sample (D3C5D+D4C5K) showed spectra that were the averages of D3ED3 and D4ED3. The monovalent samples (D3C5D+D3C5K and D4C5D+D4C5K) exhibited spectra identical to the respective untagged D3ED3 and D4ED3 spectra (**Figure 3**). Altogether, C5D and C5K tags could form both homo and hetero-oligomeric D3ED3 and D4ED3 in essentially a native-like conformation, unlike the previously reported C4I that oligomerized ED3 in a partially unfolded state due to the reverse hydrophobic effect (20, 49).

### *In Vivo* Immune Response Generated by Sub-Visible Aggregates of Bivalent and Monovalent ED3s Detected by ELISA

**Figure 5:**
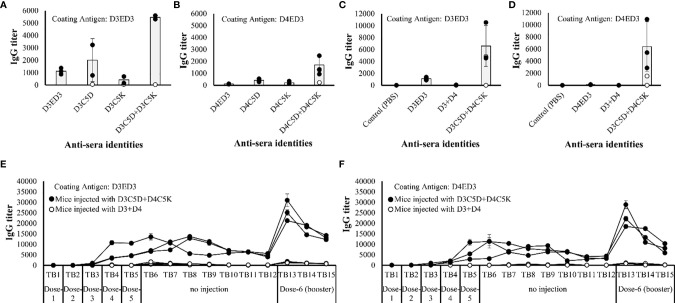
Immune response against ED3 variants. IgG titers of **(A)** D3ED3, D3C5D, D3C5K and D3C5D+D3C5K and **(B)** D4ED3, D4C5D, D4C5K and D4C5D+D4C5K after the 5^th^ dose measured using the tail-bleed sera of mice. D3ED3 and D4ED3 proteins were used as coating antigens for **(A, B)**, respectively. All mice are shown in the figure, and the total number of mice (*n*) used for immunization with the untagged and single charged ED3 mutants was *n*=3; D3C5D+D3C5K/D4C5D+D4C5K, *n*=5; D3C5D+D4C5K, *n*=5, and D3+D4, *n*=3. The average IgG titer (grey bars) was calculated using data from three highest responsive mice (closed circles), and excluding the low and non-responsive/non-bivalent ones (open circle). Antibody titers of D3+D4 and D3C5D+D4C5K (after the 5^th^ dose) when the plates were coated with **(C)** D3ED3 and **(D)** D4ED3. Long term IgG titers of D3C5D+D4C5K and D3+D4 against the coating antigens **(E)** D3ED3 and **(F)** D4ED3. “TB” indicates the number of tail-bleeding experiment counting from the first tail-bleeding after the 1^st^ injection. A booster dose was given two days after TB-12. The IgG titer of TB-13 indicates the immune response a week after the booster dose was administrated. Doses were formulated at a final protein concentration of 0.3 mg/ml in PBS or PB. Line symbols are explained within the panels.

The effects of C5D and C5K favored sub-visible aggregates of ED3s on the immunogenicity of templates D3ED3 and D4ED3 were examined in mice models. We injected five doses at one week interval as described in our previous study (20). Two doses did not induce an immune response. The IgG levels started to increase after dose-3 (**Supplementary Figures S2A–D**). After dose-5, the titer differences among mice were clearly observable. The untagged-D3ED3 was moderately immunogenic, while the D4ED3 was barely immunogenic (**Figure 5** and **Supplementary Table S1**) as assessed by anti-ED3 IgG antibody detected using ELISA. The immunogenicity (namely, anti-D3ED3 IgG antibody titers) of the monovalent combination of D3C5D+D3C5K was ~5 times higher than that of the untagged-D3ED3 (**Figure 5A**, **Supplementary Figure S2A**, and **Table S1**); and the immunogenicity of D4C5D+D4C5K was ~15 times higher than that of the untagged D4ED3 (**Figure 5B**, **Supplementary Figure S2B** and **Table S1**). On the other hand, the immune responses against the individual and thus monomeric D3C5D, D3C5K, D4C5D and D4C5K were close to that against the untagged ED3s (**Figures 5A, B** and **Supplementary Table S1**).

Noteworthy, the anti-ED3 IgG antibodies generated by the heterotypic D3C5D+D4C5K produced anti-sera with almost identical anti-D3ED3 and anti-D4ED3 titers in 3 of 4 responsive mice (**Figure 5**; **Supplementary Figure S2** and **Tables S1, S2**), and the titer was >100 fold higher than those generated by the untagged D3+D4 formulation (**Figures 5C, D** and **Supplementary Table S1**). In addition, the antibody responses of D3ED3 and D4ED3 are sero-specific with minimal or no sero-cross-recognition (20, 43). Namely, anti-D3ED3 recognizes only D3ED3 and anti-D4ED3 recognizes only D4ED3. Therefore, we concluded that the bivalent anti-D3ED3 and anti-D4ED3 raised by D3C5D+D4C5K injection originated from the bundling of the two proteins.

### T-Cell Memory Generated by Sub-Visible Aggregates of Bivalent ED3

**Figure 6:**
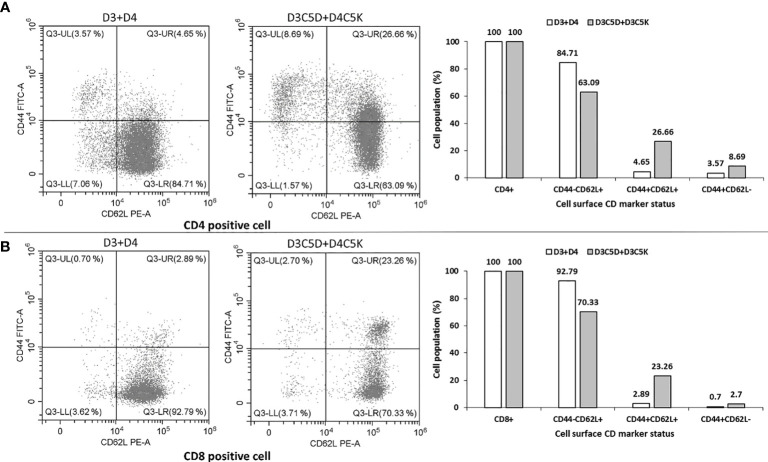
Flow cytometric analysis of the cell surface CD marker. Single cell suspension of splenocytes (obtained from a randomly selected mouse out of three that induced bivalent immune response) was used for flow-cytometry analysis by formulating into FACS buffer. Co-expression pattern of CD44 and CD62L on T-helper cell (CD4 positive) and T-cytolytic Cell (CD8 positive are shown in panel **(A, B)**, respectively. Heterotypic oligomer of tagged ED3 (D3C5D+D4C5K) induced higher proportion of CD44+CD62L+ and CD44+CD62L- co-expressed cells than that of the untagged mixture of ED3 (D3+D4).

The anti-IgG antibody titers remained high for several weeks (**Figures 5E, F**), and a booster dose of D3C5D+D4C5K generated a robust immune response against both D3ED3 (**Figure 5E**) and D4ED3 (**Figure 5F** and **Supplementary Table S1**). We then assessed the generation of immunological memory in mice immunized against D3C5D+D4C5K by analyzing the cell surface CD markers [CD4 (T-helper) and CD8 (T-cytolytic); *n=3*] 15 weeks after the first immunization). We used a randomly selected mouse for the flow cytometry experiments. The mouse injected with untagged ED3s (D3+D4) produced a high percentage of CD44-CD62L+ co-expressed CD4 (85%) and CD8 (93%) cells, indicating their naïve immunological status. On the other hand, immunization with heterotypic SCP-tagged ED3s (D3C5D+D4C5K) showed a low number of CD44-CD62L+ (on both CD4+ and CD8+ cell) when compared with those observed following D3+D4 co-immunization (**Figure 6**). These observations indicate that a central and effector T-cell memory was established through D3C5D+D4C5K immunization.

## Discussion

Oligomerization or aggregation of antigens is thought to increase a protein’s immunogenicity (14–16) through the presentation of repetitive copies of antigens (12, 32, 50). It is also well known that electrostatic interaction can cause protein oligomerization or aggregation (51, 52), but the effects of aggregates produced through electrostatic interactions on immunogenicity have barely been investigated (51, 52). Here, we generated sub-visible aggregates of both homo- and hetero- oligomeric D3ED3 and D4ED3 in a stable and reliable manner. They were most likely induced by electrostatic interactions between the lysine and aspartic acid tags and showed their contributions in increasing ED3s immunogenicity.

Antibodies directed against ED3s are effective to reduce the viral load and provide protective immunity against the infection. However, purified recombinant dengue ED3s, especially D4ED3 are poorly immunogenic (53). Phylogenetic analysis of the dengue viruses reveal some significant antigenic differences between DENV4 and the other serotypes. However, the “structural basis” for the low immunogenicity of DENV4-ED3 remains to be fully rationalized (54).

Thus, increasing the immunogenicity of poorly immunogenic ED3s is essential for vaccine development. For example, carrier proteins, lipoproteins, and fusion proteins have been proposed for increasing the anti-IgG ED3 levels in mice models, though their effect on the structure and conformation of ED3 remain to be assessed (18, 54–56). The advantage of the charged SCP-tags is that the native structures of ED3s remain unaffected as assessed by CD and fluorescence spectroscopy under the immunization conditions. In addition, it should be noted that the anti-IgG antibodies recognized ED3 itself and not the SCP-tag residues in line with our previous reports (20, 43).

We started our experiment with both D3C5D+D4C5K and D3C5K+D4C5D. While formulating D3C5K+D4C5D in PBS, pH 7.4 (same as D3C5D+D4C5K), we found that it formed tiny aggregates of ~6 nm (unpublished data). Namely, the tags interacted but not as strongly as those of D3C5D+D4C5K. Thus, we experimented at a higher pH (Tris-HCl, pH 8.8) near the pI of D3C5K+D4C5D (theoretical pI 8.25) to see whether it could form aggregates of a size similar to D3C5D+D4C5K. Eventually, we found that the proteins precipitated under that condition, and no biophysical examination could be performed. This was the rationale to proceed with the D3C5D+D4C5K and not D3C5K+D4C5D. However, in the future, it would be interesting to examine the size-dependent immunogenicity of such hetero-antigen.

A notable result was that the combination of charged SCP-tags (D3C5D+D4C5K) increased the immunogenicity of D4ED3, which is difficult to achieve with an Ile-tag (unpublished data), although Ile-tagged D3ED3 (D3C4I) increased the anti-ED3 IgG levels by thirty times (without adjuvant) with T-cell memory (20). On the other hand, a simple equimolar mixing of C5D and C5K tags attached to D3ED3 and D4ED3 favored oligomers of 500 nm, which we hypothesize increased the immunogenicity of D4ED3. To this respect, D3C5D+D4C5K produced anti-sera with strong titers against both D3ED3 and D4ED3, which were over 100 fold stronger than those generated by the untagged D3+D4. Furthermore, long-term immune response with immunological memory was induced by D3C5D+D4C5K. We assume that the strong antibody titers for both D3ED3 and D4ED3 is due to the equi-level exposure of D3ED3 and D4ED3 epitopes in the D3C5D+D4C5K heterodimer. The immune responses of the individual tagged ED3s were similar to those of the untagged ED3 proteins. C5K increased protein solubility more than C5D (26, 36), and this higher solubility possibly lowered the immunogenicity of D3C5K (57).

Altogether, in order to improve the interaction between two antigens, one can use SCP-tags to create a variety of combinations while maintaining the native proteins’ structure and conformation. At this point, it is worth reiterating that our goal was to generate an immunogenic bivalent dengue antigen using SCP-tags.

Finally, let us note that many studies dealing with aggregation and immune response show some ambiguities in correlating the aggregates’ features with immunogenicity (14–16). One reason, among others, might be the accidental formation or absence of sub-visible aggregates because they can occur through minute variation of the external conditions (31, 32). In order to alleviate such ambiguity, we measured the sizes of the sub-visible aggregates in a near-real-time manner, *i. e*., just before immunization. This near-real-time monitoring was performed in order to confirm that the *R*_hs_ remain unchanged from dose to dose and thus that no accidental aggregates formed during the experiments.

## Conclusions

This is, to the best of our knowledge, the first report showing that a mere 5-residue Asp and Lys tags can produce electrostatic interaction between two different serotype-ED3s and, therefore, enable controlling the aggregates’ size in a rational and stable manner. These sub-visible aggregates can be used to modulate the immunogenicity of ED3. Notably, the D3C5D+D4C5K formulation increased the anti-D3ED3 and anti-D4ED3 titers over 100 fold and to an equi-level. In addition, the immune response lasted for an extended period, and immune memory was observed. As of now, our goal was to report a new strategy for forming heterodimer and improving the immunogenicity of poorly immunogenic recombinant proteins. To date most other methods for sub-visible aggregate formation may affect structural integrity and/or stability, which remains unaffected using the current approach. Altogether, charged SCP-tags may provide an advantageous way to increase the immunogenicity of ED3 in both a monovalent and bivalent manner, which may provide a biotechnological platform for designing a tetravalent dengue vaccine.

## Data Availability Statement

The original contributions presented in the study are included in the article/**Supplementary Material**. Further inquiries can be directed to the corresponding author.

## Ethics Statement

The animal study was reviewed and approved by Tokyo University of Agriculture and Technology.

## Author Contributions

YK, NR, and SM designed the project. NR wrote the manuscript with YK. NR, SM, MO, MGK, and MMI performed the experiments. NR, SM, MO, and MGK analyzed and compiled the data. All authors contributed to the article and approved the submitted version.

## Funding

This study was supported by a JSPS Grant-in-Aid for Scientific Research (KAKENHI-15H04359 and 18H02385) to YK, and a Japanese government (*Monbukagakusho*: MEXT) Ph.D. scholarship to NR.

## Conflict of Interest

The authors declare that the research was conducted in the absence of any commercial or financial relationships that could be construed as a potential conflict of interest.

## Abbreviations

SCP-tags, Solubility controlling peptide tags; we use “aggregates” and “oligomers” interchangeably; Sub-visible aggregates/oligomers, Aggregates/oligomers <1000 nm that are not visible to the naked eye and remain in the soluble fraction; ED3, Envelope protein domain 3; D3 and D4 stand for Dengue virus serotypes 3 and 4, respectively; D3ED3/D4ED3, ED3 obtained from D3 or D4 serotype; D3C5X/D4C5X, D3ED3 or D4ED3 tagged with 5-Aspartic or 5-Lysine residues at their C-terminus; D3+D4, Sample containing an equimolar concentration of D3ED3 and D4ED3; D3C5D+D3C5K, Sample with an equimolar concentration of D3C5D and D3C5K; D4C5D+D4C5K, Sample with an equimolar concentration of D4C5D and D4C5K; D3C5D+D4C5K, Sample with equimolar concentration of D3C5D and D4C5K.
