# Supplementary material for: Solubility Controlling Peptide Tags of Opposite Charges Generate a Bivalent Immune Response Against Dengue ED3 Serotypes 3 and 4
Source: Front Immunol. 2021 Jun 11;12:671590. doi: 10.3389/fimmu.2021.671590 (PMC8226127; doi:10.3389/fimmu.2021.671590)
Supplement: Supplementary file 1 [file DataSheet_1.docx]

**Supporting Information**

**Solubility Controlling Peptide tags of opposite charges generate bivalent immune response against dengue ED3 serotypes 3 and 4**

Nafsoon Rahman^1$#^, Shiho Miura,^1$^ Mami Okawa^1$^, Md. Golam Kibria, Mohammad Monirul Islam^2^ and Yutaka Kuroda^1#^

^1^Department of Biotechnology and Life Sciences, Graduate School of Engineering, Tokyo University of Agriculture and Technology, 2-24-16 Naka-cho, Koganei-shi, Tokyo 184-8588, Japan. ^2^Department of Biochemistry and Molecular Biology, University of Chittagong, Chittagong-4331, Bangladesh. ^#^ Present address: Department of Biochemistry and Molecular Biology, Jagannath University, 9-10 Chittaranjan Ave, Dhaka-1100, Bangladesh

**Figure and Table legends**

**Figure S1. Effect of SCP-tags on sub-visible aggregates’ sizes measured by DLS and SLS.** A) Hydrodynamic radii of untagged and tagged ED3s including their different combinations in PBS, pH 7.4, except D3C5D+D3C5K and D4C5D+D4C5K, which were formulated in phosphate buffer (PB, pH 7.0) B) Aggregation intensities of D3ED3, D4ED3, D3C5D, D4C5K, D3+D4 and D3C5D+D4C5K in PBS at 25 °C measured by SLS C). Stability of sub-visible aggregate’s size of D3C5D+D4C5K measured over time at (C) 25° and (D) 37 °C after incubation at 25° for 20 minutes. The *R*_h_ were computed from DLS’s number spectra. Proteins were formulated at 0.3 mg/mL concentrations. Values are shown as the average of three independent measurements and three accumulations for DLS and SLS, respectively. Line symbols are explained within the panels.

**Figure S2. Immunogenicity against the sub-visible ED3 aggregates**. Dose-dependent IgG titers of A) D3C5D+D3C5K (coating antigen-D3ED3) and B) D4C5D+D4C5K (coating antigen-D4ED3) were measured using the tail-bleed sera of mice. Antibody titers of D3+D4 and D3C5D+D4C5K using C) D3ED3 and D) D4ED3 as coating antigens. Absorbance (492 nm) of anti-sera of D3+D4 and D3C5D+D4C5K (after the 5^th^ dose) using E) D3ED3 and F) D4ED3 as coating antigens. Mice identities were indicated by a, b, and c. Doses were formulated at a final protein concentration of 0.3 mg/ml. Line symbols are explained within the panels.

**Table S1: Average IgG titers of the tagged ED3s and their aggregates after 5^th^ dose and booster dose (6^th^ dose).** The titers were calculated using a power fitting model, and the values were averaged using the number of the three highest responsive mice in respective groups. Here, (-) indicated the absence of sero-cross reactive antibodies of the respective untagged and tagged ED3s as we reported ED3s’ sero-specificity [20,40] in our previous report that was also unaffected by the SCP-tags [20]. Long term immunization experiment was conducted for the mixed samples only, and therefore, not applicable (N/A) for other variants,

**Table S2: Number of total mice and corresponding responsive mice per immunization group.**

**Figure S1**


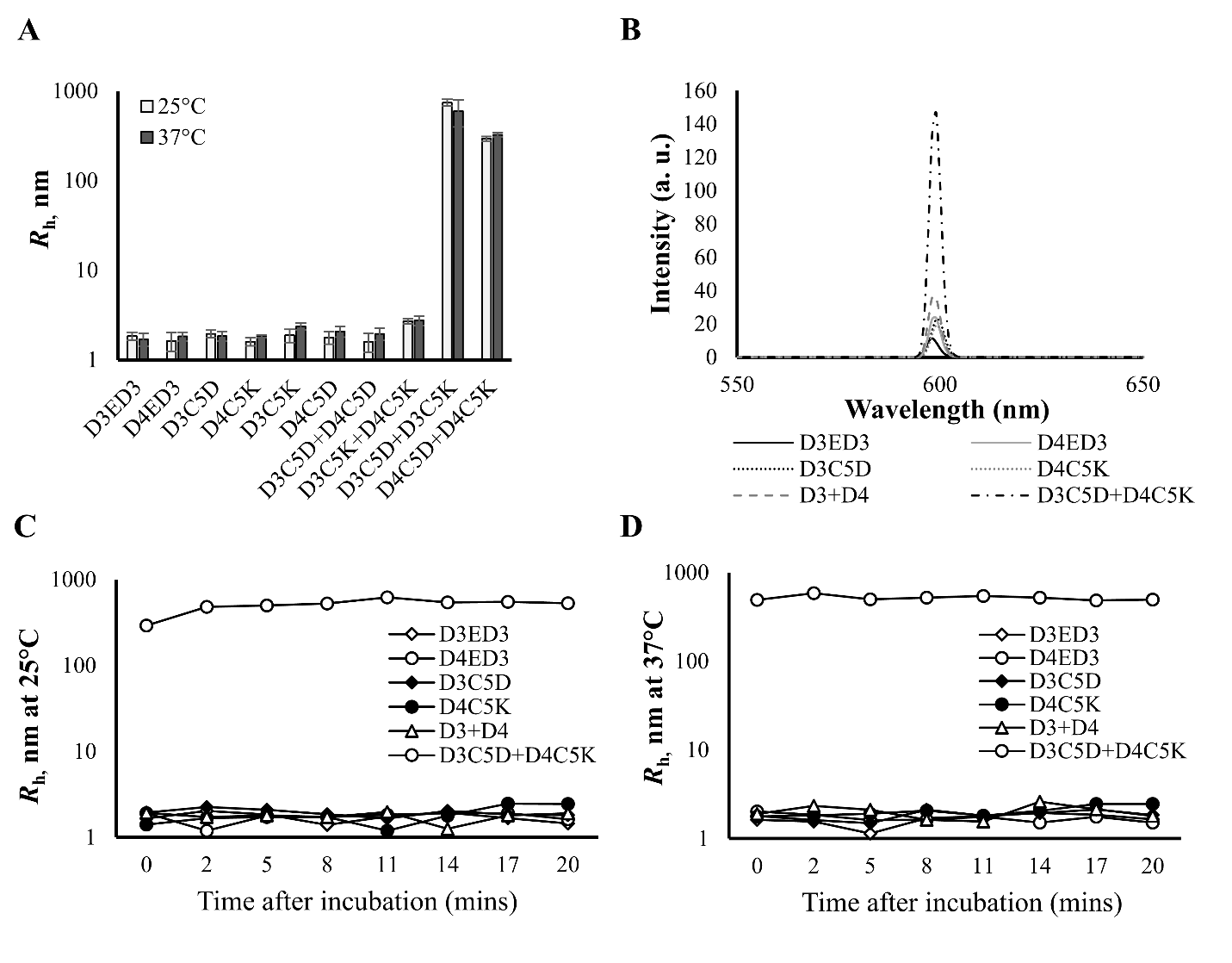


**Figure S2**


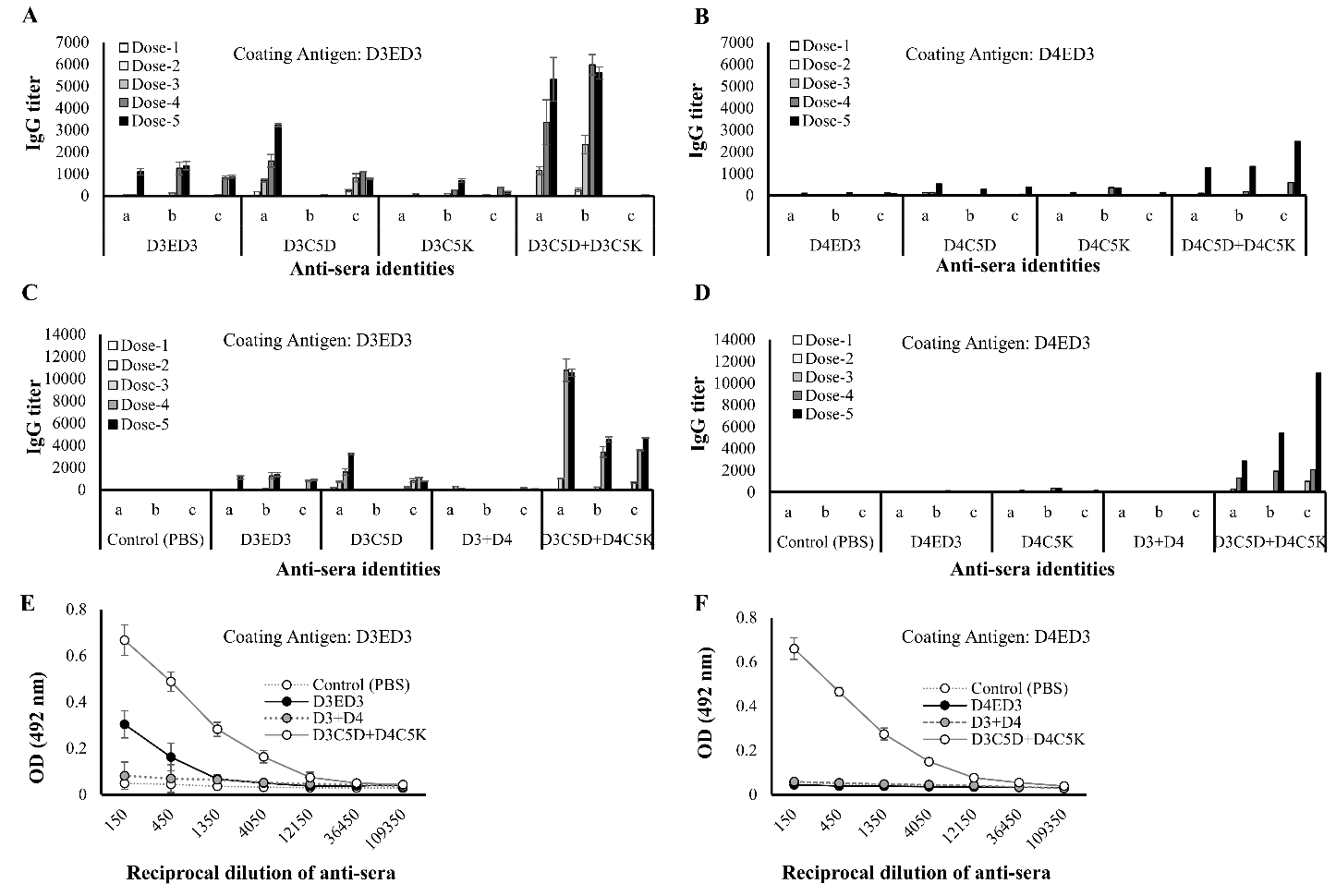


**Table S1. Average IgG titers of the tagged ED3s and fold-increase of their antibody titers after 5^th^ dose and booster dose (6^th^ dose)**

| **Protein identities** | Average IgG titer against D3ED3 (5^th^ dose) | Average IgG titer against D4ED3 (5^th^ dose) | Average IgG titer against D3ED3 (after booster dose) | Average IgG titer against D4ED3 (after booster dose) |
| --- | --- | --- | --- | --- |
| D3ED3 | 1113.8±253.4 | - | N/A | N/A |
| D3C5D | 2009.2±1732.8 | - | N/A | N/A |
| D3C5K | 423.2±377.7 | - | N/A | N/A |
| D3C5D+D3C5K | 5456.8±206.8 | - | N/A | N/A |
| D4ED3 | - | 110.6±20.6 | N/A | N/A |
| D4C5D | - | 409.5±128.7 | N/A | N/A |
| D4C5K | - | 216.4±115.5 | N/A | N/A |
| D4C5D+D4C5K | - | 1696.8±676.5 | N/A | N/A |
| D3+D4 | 35.5±40.6 | 3.3±1.3 | 1545±317.2 | 919.2±177.4 |
| D3C5D+D4C5K | 6579.1±3428.2 | 6399.7±4119.9 | 25786.9±4896.2 | 23196.3±5274 |

**Table S2. Number of total mice and corresponding responsive mice per immunization group.**

| **Immunization sample** | **Number of mice (n)** | **% of responsive mice** |
| --- | --- | --- |
| D3ED3 (coat-D3ED3) | 3 (3) | 100 |
| D4ED3 (coat-D4ED3) | 3 (3) | 100 |
| D3C5D (coat-D3ED3) | 2 (3) | 66 |
| D3C5K (coat-D3ED3) | 2 (3) | 66 |
| D4C5D (coat-D4ED3) | 3 (3) | 100 |
| D4C5K (coat-D4ED3) | 3 (3) | 100 |
| D3C5D+D3C5K (coat-D3ED3) | 2 (5) | 40 |
| D4C5D+D4C5K (coat-D4ED3) | 5 (5) | 100 |
| D3C5D+D4C5K (coat-D3ED3) | 4 (5)^a^ | 80^a^ |
| D3C5D+D4C5K (coat-D4ED3) | 4 (5)^a^ | 80^a^ |
| D3+D4 (coat-D3ED3) | 1 (3) | 33 |
| D3+D4 (coat-D4ED3) | 0 (3) | 0 |

^a^Among the 5 mice of D3C5D+D4C5K, only 4 (80%) was responsive though 1 was non-bivalent (not identical recognition of D3ED3 and D4ED3) i.e. the percentage of bivalent mice was 75%
